# Validation of an instrument for the assessment of patient-centred care among patients with multimorbidity in the primary care setting: the 36-item patient-centred primary care instrument

**DOI:** 10.1186/s12875-018-0832-4

**Published:** 2018-08-28

**Authors:** Jane Murray Cramm, Anna Petra Nieboer

**Affiliations:** Erasmus School of Health Policy & Management, Erasmus University, Rotterdam, The Netherlands

**Keywords:** Patient-centred care, Multimorbidity, Primary care, Instrument development, PCPC instrument

## Abstract

**Background:**

Validated instruments are needed to assess the delivery of patient-centred care (PCC) to patients with multimorbidity in the primary care setting. Eight dimensions of PCC have been identified: respect for patients’ preferences, access to care, emotional support, information and education, involvement of family and friends, continuity and secure transition between health care settings, physical comfort, and coordination of care. The main objective of this study was to validate an instrument for the assessment of PCC among patients with multimorbidity in the primary care setting: the 36-item patient-centred primary care (PCPC) instrument.

**Methods:**

We included patients with multimorbidity from seven health care practices in the region of Tilburg, the Netherlands. All patients enrolled in at least two chronic care programmes (involving diagnosis of and treatment for combinations of diabetes, asthma and/or chronic obstructive pulmonary disease, cardiovascular diseases and conditions, and age-related frailty) were selected from the practices’ data registries and included as eligible participants. A total of 216 patients with multimorbidity filled in the study questionnaire (55% response rate). We tested the instrument using structural equation modelling, and examined its validity and reliability.

**Results:**

Confirmatory factor analyses revealed good indices of fit and overall internal consistency, as represented by Cronbach’s alpha values. All eight dimensions of PCC were related positively to satisfaction with care (all *p* ≤ 0.001). Patients with multimorbidity who experienced joint decision making and responsibility taking in the primary care setting also had significantly higher scores for all eight PCC dimensions, indicating the instrument’s construct validity.

**Conclusions:**

We conclude that the psychometric properties of the 36-item PCPC instrument are good. Based on these results the PCPC instrument seems a promising tool for the assessment of PCC among patients with multimorbidity in the primary care setting.

## Background

The number of people with chronic diseases is increasing at an astonishing rate, making millions of patients reliant on primary care systems. As a consequence, such systems throughout the world are struggling to find the best way to deal with large numbers of chronically ill patients while keeping costs low and quality high [1]. Patients with multimorbidity are known to report even worse well-being and health outcomes than do individuals with single chronic conditions [2]. Furthermore, they are more likely to be hospitalised and are at greater risk of premature death than are patients with single chronic diseases [3, 4]. The prevalence of multimorbidity in Europe is currently about 65% among people aged ≥60 years and 85% among those aged ≥85 years [5–7]. Thus, 50 million people in Europe have at least two chronic diseases, and this number is expected to grow as populations age.

Providing optimal care for older patients with (multiple) chronic diseases is actually among the greatest challenges of the healthcare spectrum today [8]. Health professionals clearly express the concern that they often missed the opportunity to engage with multimorbidity patients as true partners in their care and really help them to manage their conditions at home. This situation represents a missed opportunity, as the outpatient primary care setting is precisely the context identified as most appropriate to effectively deal with ageing populations [6–8]. The complexity of many chronic diseases and frailty profiles that come with multimorbidity demands Patient Centred Care (PCC), such that multimorbidity patients are equipped with the information and skills necessary to act as co-producers or co-creators of their care and where abilities to maintain overall well-being are optimised [7–9]. Such care supports active involvement of patients in the design of new care models and in decision making about individual treatment options. Decisions need to be based on personal preferences, needs and circumstances of each patient in every aspect of their live beyond physical health and clinical outcomes only [9–13].

The Harvard School of Medicine collaborated with the Picker Institute to conduct a very comprehensive study of the constituents of PCC [14, 15]. These researchers identified the following eight dimensions of PCC: respect for patients’ values, preferences and expressed needs, provision of information and education, access to care, emotional support to relieve fear and anxiety, involvement of family and friends, continuity and secure transition between health care settings, physical comfort, and coordination of care [14–16]. A consultation of additional PCC literature [17–21] revealed no additional aspect or dimension of PCC. Table 1 provides detailed descriptions of the eight PCC dimensions in general [14–21] and describes them for patients with multimorbidity receiving primary care in particular as derived from the literature [22–25].

**Table 1:** The eight dimensions of patient-centred care in the primary care setting for patients with multimorbidity

Dimension	Description
1) Patients’ preferences	Patients with multimorbidity have indicated that they need to be seen as a whole person instead of a sum of certain diseases. It is, for example, not sufficient to complete a standard template of a care plan for a patient with COPD and another template for the same patient’s diabetes. Whole-person care is a concept requiring professionals’ understanding of each patient as a whole by taking the time to really get to know the patient and his/her values and preferences, thereby improving the patient’s well-being. To enhance PCC, health care professionals should involve chronically ill and multimorbidity patients in decisions about their care and support them in setting and achieving their own treatment and life goals. Preference-sensitive decisions include therapy that may improve one condition but make another worse (e.g., corticosteroids for chronic obstructive pulmonary disease may exacerbate osteoporosis); therapy that may confer long-term benefits but cause short-term harm (e.g., preventive agents, such as statins, frequently have adverse effects); and multiple medications, each with benefits and harms that must be balanced. Finally, preferences may change over time and should be re-examined, particularly with a change in health status.
2) Information and education	The provision of complete information to patients about all aspects of their care is necessary. Patients should have access to their care records (e.g. via e-health applications) and be in charge of their care. Open communication between patients and health care professionals, which requires professionals to possess high-quality communication skills, is also necessary. Although healthcare professionals may often feel that some adverse effects are less important than expected benefits, patients with multimorbidity often consider them to be highly significant given that they often use multiple medicines and treatments. Multimorbidity disproportionally affects those from lower socio-economic groups, which calls for information suitable for patients from all educational levels.
3) Access to care	Access to care refers to waiting times (to schedule an appointment as well as waiting time during the visit), an accessible building (including those with physical limitations and mobility problems) as well as medicine access (patients can easily request a repeat recipe). Research unfortunately shows older patients with (multiple) chronic diseases experience many difficulties when it comes to access to care and find it difficult to make an appointment with their general practitioner and specialist. Obstacles include overloaded telephone lines and the inability to schedule an appointment less than several months in advance. Furthermore, special attention is needed for (im)migrant and illiterate patients.
4) Emotional support	Multimorbidity patients often experience anxiety about the impact of their multiple illnesses on their lives as a whole not just their physical health outcomes. Being in constant pain and feeling tired also limits the ability to invest in social relationships and to keep your job, which may cause a lot of worries and anxieties. Proper support is needed to help patients with multimorbidity in their abilities to achieve social and mental well-being, which is currently a major challenge in the primary care setting.
5) Family and friends	In the case of chronical diseases and especially multimorbidity (depending on the seriousness of the conditions), these illnesses not only affect the patient, but also his/her family and friends. In such cases, PCC may be improved by the involvement of relatives in decisions about the patient’s care, and attention to the role and needs of informal caregivers. Furthermore, optimal care of older adults with multimorbidity is best achieved by a collaborative effort that involves patients, family members, and health care providers. Family members, especially spouses, often play a leading role in sharing responsibility for some of the care. A person-centred approach begins by gathering specific information about a person’s preferences in light of health circumstances, with input from family members and other caregivers if the person wishes. Added to a comprehensive health and functional assessment within the patient preferences dimension, this information is used to help a person shape and articulate his or her health and life goals. These goals are driven first and foremost by how a person wants to function and what he or she envisions for future well-being.
6) Continuity and transition	Continuity and secure transition between health care settings have been identified as important aspects of PCC for patients with multimorbidity. Smooth transitions require the transfer of all relevant patient information; ensuring that patients are well informed about where they are going, what care they will receive, and who their contact person will be; and the provision of skilled advice about care and support at home after discharge from a hospital for example. Involvement of various healthcare professionals, numerous treatments and taking multiple medications is known to have adverse effects. A complex regimen is associated with non-adherence, adverse drug events, economic burden, and informal caregiver stress. These risks can worsen with impairments in cognitive and physical function. Thus, it is recommended to periodically evaluate the patient’s capability to manage medications. Continuity and smooth transition can help simplifying the regimen as well as carefully monitoring the patient with feedback if needed. Older patients with more complex health and psychosocial issues indeed are known to benefit from comprehensive evaluation by geriatricians, psychiatrists, social workers, and home care providers, who continuously work well together.
7) Physical comfort	Multimorbidity patients’ physical comfort should be supported effectively in order to support their ability to achieve physical well-being. Pain should be managed effectively, patients have to sleep well and healthcare professionals should take patients’ needs about support and their daily living needs into account. Not just physical comfort in the daily life of patients matters, but they should also be provided with comfort during their visit to the healthcare professional; areas should for example be clean and comfortable and patients’ privacy must be respected.
8) Coordination of care	Patient care should be well coordinated among professionals (teamwork in care delivery). Health care professionals should be well informed so that patients do not have to repeat their stories over and over again. Given the involvement of multiple professionals in the case of patients with multimorbidity, this becomes increasingly important.

*PCC* patient-centred care

In a systematic review, Rathert and colleagues [16] showed that organisations which perform well in multiple PCC dimensions report better organisational and patient outcomes (e.g. improved care processes, clinical outcomes, cost reduction). For example, care that is well coordinated, easily accessible, providing physical comfort and attending patients’ emotional needs is known to increase patients’ satisfaction with care as well as improve joint decision making and responsibility taking [16, 26–29]. Organisations implementing interventions falling into several PCC dimensions reported better outcomes than did those aiming to improve single PCC dimensions, indicating that a constellation of interventions in multiple PCC dimensions leads to better outcomes [16].

Although we have considerable evidence for the benefits of investing in the improvement of the eight PCC dimensions [14–16] to achieve better organisational and patient outcomes, we lack instruments and research investigating the effects of these eight dimensions, especially among patients with multimorbidity in the primary care setting. A broader understanding of patients’ experiences with these PCC aspects is important as they could help to improve the organisation and provision of care for this population, which is expected to lead to better outcomes (e.g. improved satisfaction with care, enhanced self-management abilities and well-being) [16, 22–25]. The measurement of levels of PCC among patients with multimorbidity is the first step. Therefore, the main objective of this study is to validate an instrument for the assessment of PCC among patients with multimorbidity in the primary care setting: the 36-item patient-centred primary care (PCPC) instrument. Based on earlier research we expected that investment in the eight dimensions of PCC would be associated positively with satisfaction with care [16]. In addition, we expected that the occurrence of joint decision making and responsibility taking, as perceived by patients with multimorbidity, would align with their experiences with PCC.

## Methods

We included adult patients with multimorbidity from seven health care practices in the region of Tilburg, the Netherlands. All patients enrolled in at least two chronic care programs (involving diagnosis of and treatment for combinations of diabetes, asthma and/or chronic obstructive pulmonary disease, cardiovascular diseases and conditions, and age-related frailty) were selected from the data registries of the practices and included as eligible participants. All adult patients with at least two registered chronic conditions (*n* = 413) were eligible to participate. No additional inclusion criterion was applied. Exclusion criteria were too ill to participate and no longer a patient of the health care practices under study. First, these selected patients received questionnaires at home via post. Three weeks later, reminder notices were sent to non-respondents. Another three weeks later, second reminder notices with duplicates of the questionnaire were sent. Finally, when telephone numbers were available, we called non-respondents to ask them to fill in the questionnaire. Nineteen respondents appeared not eligible to participate due to incorrect addresses (*n* = 5), recent moves (*n* = 2), death (*n* = 4), admission to a hospice or nursing home due to terminal illness (*n* = 2), poor cognitive function preventing questionnaire filling (*n* = 2), recent stroke (*n* = 1), and poor eyesight (*n* = 3). Of the remaining 394 patients a total of 216 patients with multimorbidity filled in the questionnaire. Thus, the final response rate was 55%.

The medical ethics committee of Erasmus Medical Centre, Rotterdam, the Netherlands determined that the rules stipulated in the Medical Research Involving Human Subjects Act did not apply to this study (protocol no. MEC-2018-021). Written informed consent to participate in the study was obtained from all participants.

### Measures

#### Patient-centred primary care

The eight dimensions of PCC identified by the Picker Institute were used as a framework for the development of an instrument to assess PCC in the primary care setting for patients with multimorbidity. The development of the patient-centred primary care (PCPC) instrument also builts on our earlier work [30–32], in which we investigated the eight dimensions of PCC in hospital and long-term care settings, as well as adjusting them based on literature describing primary care for patients with multimorbidity (e.g. [22–25]) and consulting experts in the primary care setting when it comes to care for patients with multimorbidity (i.e., general practitioners, practice nurses, and patients with multimorbidity, who were consulted on item adjustment, removal, and addition). General practitioners (*n* = 4) and practice nurses (*n* = 3) known for delivering high levels of PCC in the area of Tilburg and patients dealing with multimorbidity (n = 3) for several years were selected as experts. First, they received the questionnaire via mail. All experts commented on the items via mail followed by a telephone meeting. Both authors discussed the comments and suggestions, which led to an adjusted version we send back to the experts. This was followed by another telephone meeting (with a selection of experts) and a personal meeting (with a patient expert) after which we reached agreement on a final set of 36 items, with responses structured by a five-point scale ranging from 1 (totally disagree) to 5 (totally agree).

#### Satisfaction with care

We used an adjusted version of the Satisfaction with inpatient Stroke Care (SASC) scale to assess satisfaction with care. Although the 8-item SASC was originally developed for use among stroke patients, it investigates experiences with care in general, not those related specifically to stroke care. Therefore, the SASC has been used widely in various patient populations to assess general satisfaction with care (e.g. [33–36]). The items were adjusted slightly for the primary care setting (e.g. ‘The doctors have done everything they can to make me well again’ was changed into ‘The staff has done everything they can to make me well again’), with removal of irrelevant or overlapping items (e.g. the hospitalization process went smoothly and I have been treated with kindness and respect by the staff at the hospital), resulting in a final set of 6 items: ‘I have received all the information I want about the causes and nature of my illness(es)’, ‘The staff has done everything they can to make me well again’, ‘I am satisfied with the type of treatment they have given me (e. g. physiotherapy, occupational therapy)’, ‘I have had enough therapy (e.g. physiotherapy, occupational therapy)’, ‘I am happy about the effect treatments had on my disease progression’, and ‘I am satisfied with the treatment provided by the general practitioner who I visit’. Responses are structured by a four-point scale ranging from 1 (strongly disagree) to 4 (strongly agree), with higher mean scores indicating greater satisfaction. In this study among patients with multimorbidity, the Cronbach’s alpha value for this adjusted 6-item instrument was 0.89, indicating good reliability.

#### Joint decision making and responsibility taking: Relational co-production of care

The 7-item relational co-production instrument was used to assess joint decision making and responsibility taking, as perceived by patients with multimorbidity [10, 37, 38]. Relational co-production refers to joint decision making and responsibility taking achieved through open communication, co-operation, and respect for each other, with negotiation of treatment options to accomplish mutually defined goals. Gittell (e.g. [39–42]) identified this concept as “relational coordination” when talking about the quality of relationships and communication among professionals and “relational coordination” if it concerns the quality of relationships and communication between patients and their health care professionals. High-quality relationships reinforce high-quality communication, encouraging professionals and patients with multimorbidity to listen to each other and to take account of the impacts of their actions on those engaged in different parts of the process, thereby helping them to react to new information in a co-ordinated way [43]. This questionnaire measures four aspects of communication (frequent, timely, accurate, and problem-solving) and three aspects of relationships (based on shared knowledge, goals, and mutual respect) between professionals and patients with multimorbidity. Responses are structured by a five-point Likert scale ranging from 1 (never) to 5 (always). This instrument has proven to be reliable and valid (based on structural and content validity, internal consistency and interrater agreement) in assessing the quality of communication and relationships [44, 45]. Higher mean scores indicate better realisation of joint decision making and responsibility taking, with scores ≥4 considered to represent success and scores < 4 considered to represent the lack of success perceived by patients with multimorbidity [46]. In this study, the Cronbach’s alpha value for this instrument was 0.87, indicating good reliability.

### Analysis

To validate the 36-item PCPC instrument, we first used descriptive statistics to characterise the study population with regard to age, gender, marital status, educational level, and experiences with primary care delivery (the eight dimensions of PCC and satisfaction with care). Second, we calculated the mean, standard deviation, number of missing responses, and lambda value for each PCPC item. Third, we used LISREL to conduct confirmatory factor analyses and verify the factor structure of the instrument. Fourth, we assessed model fit using the following cut-off criteria of Hu and Bentler [47]:

- standardised root mean square residual (SRMR) < 0.08,
- root mean square error of approximation (RMSEA) < 0.06, and
- comparative fit index (CFI) > 0.95.

Fifth, we used Cronbach’s alpha values to assess the internal consistency of the subscales and examined inter-correlations to verify conceptual relatedness among (sub)scales. Finally, we assessed the construct validity of the instrument overall and its eight dimensions by analysing associations with satisfaction with care. In addition, we examined whether scores for the eight dimensions of PCC were higher among patients who perceived the successful establishment of joint decision making and responsibility taking with their health care professionals than among those who perceived a lack of success.

## Results

The mean age of the patients was 74.46 ± 10.64 (range 47–94) years (Table 2). Of the respondents, 40.9% were male, 43.2% were single, and 40% had lower educational levels.

**Table 2:** Characteristics of patients with multimorbidity in the study sample and their experiences with care

Characteristic		Percentage or mean (SD) range
Gender	Male	40.9%
Marital status	Single	43.2%
Educational level	Low	40.0%
Age (years)		74.46 (10.64) 47–94
Patient-centred care	Patients’ preferences	3.94 (0.64) 1–5
	Physical comfort	3.91 (0.56) 1–5
	Coordination of care	3.89 (0.61) 1–5
	Emotional support	3.43 (0.75) 1–5
	Access to care	4.03 (0.56) 1–5
	Continuity and transition	3.97 (0.58) 1–5
	Information and education	3.89 (0.56) 1–5
	Family and friends	3.62 (1.00) 1–5
	Overall patient-centred care	3.83 (0.47) 1–5
Satisfaction with care		3.13 (0.45) 1–4

Results are based on list-wise deletion of missing cases. Results based on imputed data (*n* = 216) were similar. SD = Standard Deviation

### PCPC item characteristics

Table 3 displays statistics for the 36 PCPC items. Item non-response rates ranged from 1 to 8%. Respondents gave considerable numbers of not-applicable responses in the family and friends dimension (about 100 per item), continuity and transition (*n* = 36–54), and coordination of care (item 14, *n* = 38; item 13, *n* = 24) dimensions. In addition, 61 respondents rated item 23 in the emotional support dimension as not applicable, and 19 and 58 respondents rated items 8 and 9, respectively, in the physical comfort dimension as not applicable.

**Table 3:** Characteristics of responses to the 36 patient-centred care items (*n* = 216)

Item	Valid *n*	Not applicable	Missing	Mean	SD	λ
Patients’ preferences						
1. I felt taken seriously	214	–	2 (1%)	4.16	0.77	0.779
2.My wishes and preferences were taken into account when choosing a treatment	209	–	7 (3%)	3.93	0.75	0.870
3.I was involved in decisions about my treatment	209	–	7 (3%)	4.01	0.74	0.854
4.The influence that the treatment can have on my life was taken into account	209	–	7 (3%)	3.92	0.81	0.894
5. I was helped to determine my own treatment goals	211	–	5 (2%)	3.82	0.78	0.877
6.I felt supported to achieve my treatment goals	209	–	7 (3%)	3.89	0.78	0.825
7. I received advice that I really could use	209	–	7 (3%)	3.90	0.85	0.869
Physical comfort						
8. Attention was given to my physical comfort (such as the management of pain, shortness of breath)	190	19 (9%)	7 (3%)	4.10	0.83	0.736
9. Attention was paid to fatigue and insomnia	148	58 (27%)	10 (5%)	3.64	0.93	0.788
10. The (waiting) rooms were clean	210	–	6 (3%)	4.13	0.68	0.650
11. The (waiting) rooms were comfortable	211	–	5 (2%)	3.85	0.77	0.528
12. In the treatment room(s) and at the counter there was sufficient privacy	211	–	5 (2%)	3.77	0.89	0.681
Coordination of care						
13. Everyone was well informed; I only had to tell my story once	189	24 (11%)	3 (1%)	3.98	0.80	0.791
14. The care was well attuned between the practitioners involved		38 (18%)	3 (1%)	3.99	0.72	0.849
15. I knew who was coordinating my care	210	–	6 (3%)	3.75	0.82	0.753
16. I could easily contact someone with questions	209	–	7 (3%)	3.88	0.74	0.874
Continuity and transition						
17. When being referred to another care provider (specialist/dietician/physiotherapist) I was well informed about where to go and why	157	54 (25%)	5 (2%)	4.05	0.72	0.727
18. With a referral, all my information was passed on correctly	163	48 (22%)	5 (2%)	4.00	0.75	0.791
19. Advice (such as medication) from different practitioners (medical specialists and family doctor) was well attuned to each other	174	36 (17%)	6 (3%)	3.92	0.79	0.741
20. The treatment of the family doctor is in line with the treatment of other care providers	169	42 (19%)	5 (2%)	3.98	0.68	0.870
Emotional support						
21. Emotional support was also provided	202	–	14 (7%)	3.47	0.84	0.831
22. Attention was paid to possible feelings of fear, gloom and anxiety	98	–	18 (8%)	3.46	0.85	0.841
23. I was made aware of the possibilities for more intensive emotional support	146	61 (28%)	9 (4%)	3.30	1.03	0.851
24. Attention was paid to the impact of my health on my private life (family, relatives, work, social life)	202	–	14 (7%)	3.43	0.83	0.921
Access to care						
25. It was no problem to go from my home to my family doctor and back again	209	–	7 (3%)	3.92	1.03	0.700
26. The general practice was easily accessible	209	–	7 (3%)	4.24	0.63	0.817
27. I could easily schedule an appointment quickly	209	–	7 (3%)	4.00	0.72	0.778
28. On a visit I didn’t have to wait long before it was my turn	206	–	10 (5%)	3.73	0.85	0.639
29. I could easily request a repeat recipe	207		9 (4%)	4.24	0.62	0.851
Information and education						
30. I was well informed	209	–	7 (3%)	4.02	0.65	0.883
31. The information I received was well explained	208	–	8 (4%)	3.99	0.67	0.889
32. I had easy access to my own data (lab results, medication overview, referrals)	203	–	13 (6%)	3.47	0.95	0.706
33. I could ask all the questions I wanted	211	–	5 (2%)	4.08	0.60	0.794
Family and friends						
34. With my consent, relatives were involved in my treatment	105	103 (48%)	8 (4%)	3.74	1.03	0.954
35. Attention was given to care and support provided by family members	101	108 (50%)	7 (3%)	3.49	1.11	0.906
36. Attention was given to possible questions from my family members	98	110 (51%)	8 (4%)	3.69	0.94	0.903

All items had loadings on the intended factors > 0.50.

### Model fit

The model showed good fit, meeting cut-off criteria (CFI = 0.987, SRMR = 0.079, RMSEA = 0.0548).

### Internal consistency and inter-correlations

Internal consistency values for the PCPC subscales ranged from 0.72 (physical comfort) to 0.92 (family and friends; Table 4). The internal consistency value for the overall instrument was 0.89. All (sub)scales were correlated significantly and positively (all *p* ≤ 0.001), indicating that they were conceptually related.

**Table 4:** Scale characteristics and (inter-)correlations of the 36-item patient-centred primary care instrument

	Cronbach’s *α*	1	2	3	4	5	6	7	8
1. Patients’ preferences	0.92								
2. Physical comfort	0.72	0.57***							
3. Coordination of care	0.82	0.60***	0.52***						
4. Emotional support	0.87	0.46***	0.37***	0.50***					
5. Access to care	0.74	0.45***	0.54***	0.53***	0.30***				
6. Continuity and transition	0.80	0.63***	0.59***	0.67***	0.43***	0.53***			
7. Information and education	0.78	0.56***	0.48***	0.57***	0.57***	0.53***	0.58***		
8. Family and friends	0.92	0.31***	0.37***	0.36***	0.43***	0.33***	0.42***	0.36***	
9. Overall patient-centred care	0.89^a^	0.77***	0.73***	0.80***	0.71***	0.68***	0.80***	0.76***	0.66***

***p < 0.001 (two-tailed). Results are based on list-wise deletion of missing cases. Results based on imputed data (*n* = 216) were similar. ^a^ Based on the eight dimensions (the value for the 36 items was 0.96)

### Construct validity

All eight dimensions of PCC were related positively to satisfaction with care (all *p* ≤ 0.001), indicating construct validity (Table 5). In addition, scores in all dimensions were higher among patients with multimorbidity who experienced joint decision making and responsibility taking (Table 6).

**Table 5:** Correlations of patient-centred care dimension scores with satisfaction with care

Dimension	Satisfaction with care
Patients’ preferences	0.45***
Physical comfort	0.38***
Coordination of care	0.49***
Emotional support	0.32***
Access to care	0.46***
Continuity and transition	0.51***
Information and education	0.46***
Family and friends	0.34***
Overall person-centred care	0.53***

***p < 0.001 (two-tailed). Results are based on list-wise deletion of missing cases. Results based on imputed data (*n* = 216) were similar

**Table 6:** Relationships between joint decision making and responsibility taking (relational co-production of care) and patient-centred care

Dimension	No joint decision making/ responsibility taking (score ≥ 4; 37%)	Joint decision making/ responsibility taking (score < 4; 63%)	*p* value
Patients’ preferences	4.16 (0.57)	3.81 (0.65)	≤0.001
Physical comfort	4.09 (0.60)	3.81 (0.51)	≤0.001
Coordination of care	4.11 (0.55)	3.76 (0.62)	≤0.001
Emotional support	3.69 (0.65)	3.30 (0.76)	≤0.001
Access to care	4.22 (0.47)	3.91 (0.57)	≤0.001
Continuity and transition	4.21 (0.55)	3.82 (0.56)	≤0.001
Information and education	4.14 (0.53)	3.82 (0.56)	≤0.001
Family and friends	3.93 (0.94)	3.41 (0.98)	0.009
Overall patient-centred care	4.05 (0.45)	3.71 (0.44)	≤0.001

Results are based on list-wise deletion of missing cases. Results based on imputed data (*n* = 216) were similar

## Discussion

This study clearly showed that the 36-item PCPC instrument is valid and reliable for the assessment of PCC among patients with multimorbidity in the primary care setting. Given that multimorbidity is becoming the leading threat to population health and the greatest challenge for primary care systems worldwide, such an instrument can help to improve levels of patient centredness for this vulnerable population. Findings from several countries indicate that the prevalence of chronic diseases and multimorbidity is especially high in the primary care setting [8, 48–51]. Primary care providers are currently not equipped to deal with the complexities of ageing populations [51]. Health professionals clearly express the concern that they often missed the opportunity to engage chronically ill older patients as true partners in their care and really help them to manage their conditions at home. Identification of levels of patient centredness according to patients with multimorbidity provides insight in how well organizations are doing regarding the eight dimensions of PCC and helps identify the areas which need improvement (the dimensions with lower scores). Confirmatory factor analyses revealed good indices of fit for the instrument. As indicated by the high reliability coefficient, the scale showed good internal consistency. We found support for construct validity through significant positive correlations between PCPC scores and satisfaction with care. These findings are in line with earlier research showing positive associations between the eight dimensions of PCC and satisfaction with care [16]. This research also showed that patients with multimorbidity who perceived joint decision making and responsibility taking with their health care professionals also reported higher levels of all eight dimensions of PCC.

Several psychometric properties of the PCPC instrument could not be evaluated in this study and thus remain undefined. They include the instrument’s responsiveness, sensitivity to change, predictive value (e.g. clinical outcomes), relationship to other PCC instruments, and different modes of administration. Secondly, we included patients enrolled in at least two chronic care programmes (involving diagnosis of and treatment for combinations of diabetes, asthma and/or chronic obstructive pulmonary disease, cardiovascular diseases and conditions, and age-related frailty). Given that these patients were enrolled in at least 2 or more chronic care programmes we were able to identify and select them from the data registries of the health care practices. As a consequence patients who are dealing with a chronic illness other than diabetes, asthma and/or chronic obstructive pulmonary disease, cardiovascular diseases and conditions, and age-related frailty (which are the most prevalent chronic conditions) were not included. Further research is needed to assess the validity of this instrument among patients with (a combination of) other chronic illnesses as well as the general patient population in the primary care setting. Thirdly, some items had high numbers of ‘non-applicable’ responses. Although these aspects may not be applicable to all patients with multimorbidity in the primary care setting (e.g., emotional support and attention paid to fatigue and insomnia is only needed for those who struggle in these areas and transition of care is only relevant for those who are referred to another health care professional), if they are applicable they are crucial for their outcomes [22–24]. Fourthly, while after four iterative rounds we reached agreement on a final set of 36 items, we did not use a formal consensus method. Fifthly, while the original SASC to assess satisfaction with care has been validated among patients in the hospital we slightly adjusted the instrument to assess satisfaction with care among patients with multimorbidity in the primary care setting (e.g. replacing ‘doctor’ with ‘staff’). Although the Cronbach’s alpha (0.89) shows the instrument is reliable in this setting this adjusted 6-item SASC has not been formally validated yet. This may have limited testing concurrent construct validity of the PCC instrument. Finally, we tested the Dutch version of the 36-item PCPC instrument; we recommend testing of the English version in other countries to ensure international validity using adequate translation procedures such as forward-translations and back-translations recommended by the World Health Organisation [52].

## Conclusion

We conclude that the psychometric properties of the 36-item PCPC instrument are good. Based on these results the PCPC instrument seems a promising tool for the assessment of PCC among patients with multimorbidity in the primary care setting.

## Abbreviations

CFI: Comparative fit index
PCC: Patient-centred care
PCPC instrument: Patient-centred primary care instrument
RMSEA: Root mean square error of approximation
SASC: Satisfaction with inpatient stroke care
SD: Standard deviation
SRMR: Standardised root mean square residual
